# Longitudinal plasma proteomics identifies diagnostic and response-associated inflammatory and immune biomarkers in psoriasis following secukinumab therapy

**DOI:** 10.3389/fimmu.2026.1806248

**Published:** 2026-04-24

**Authors:** Hongyang Zhang, Rui Yang, Yu Ma, Jiahui Yang, Binyan Yang, Lingdi Dong, Nan Yu

**Affiliations:** 1 Department of Dermatology, General Hospital of Ningxia Medical University, Yinchuan, Ningxia, China; 2 First Clinical College of Ningxia Medical University, Yinchuan, Ningxia, China

**Keywords:** psoriasis, secukinumab, IL-17 signaling, plasma proteomics, response biomarkers

## Abstract

**Introduction:**

Psoriasis (PSO) is a chronic immune-mediated inflammatory skin disease characterized by keratinocyte hyperproliferation and dysregulated activation of the IL-23/IL-17 axis, leading to persistent cutaneous inflammation and substantial disease burden. Comprehensive profiling of circulating inflammatory proteins provides important insights into the systemic immune alterations associated with PSO and its treatment. However, the longitudinal plasma proteomic changes induced by IL-17A blockade remain incompletely characterized.

**Methods:**

We quantified the expression levels of 92 inflammation-related proteins in plasma samples from 10 patients with moderate-to-severe PSO before and during secukinumab therapy, as well as from 10 healthy controls (NC), using the Olink Target 96 Inflammation panel. Key differentially expressed proteins were further validated by enzyme-linked immunosorbent assay (ELISA). Longitudinal patterns were assessed using Mfuzz clustering, and functional enrichment analyses were performed to explore the underlying biological pathways.

**Results:**

Baseline plasma proteomic profiles clearly discriminated PSO patients from healthy individuals, with CXCL1, CXCL5, CCL20, and HGF exhibiting the most pronounced alterations and the highest diagnostic performance. Longitudinal analysis revealed a coordinated reduction of multiple inflammatory mediators following IL-17A inhibition, including CCL20, IL-17C, IL-6, and OSM. Mfuzz analysis identified two dominant temporal expression patterns characterized by a progressive decrease in inflammatory and immune-related proteins over the course of secukinumab treatment. Notably, baseline circulating IL-17C levels were significantly positively correlated with disease severity, suggesting its potential as a biomarker of therapeutic response. Functional enrichment analysis indicated that differentially expressed proteins (DEPs) were mainly involved in cytokine–cytokine receptor interaction and IL-17-related signaling pathways.

**Discussion:**

To our knowledge, this is the first study to characterize the longitudinal plasma proteomic landscape of PSO patients treated with secukinumab using the Olink platform. This work provides molecular evidence for the systemic immunomodulatory effects of IL-17A blockade and highlights candidate circulating biomarkers for disease monitoring and treatment response in PSO. Given the small sample size, these results should be validated in larger, independent cohorts.

## Introduction

PSO is a systemic, immune-mediated inflammatory disease primarily driven by aberrant activation of the interleukin-23/interleukin-17 (IL-23/IL-17) axis (1, 2). Beyond cutaneous involvement, patients with PSO often display widespread systemic inflammatory responses, as evidenced by altered levels of multiple immune- and inflammation-related factors in the peripheral circulation (3, 4). These molecular changes not only reflect disease activity but are also closely associated with metabolic and cardiovascular comorbidities (5). Interleukin-17A inhibitors, such as secukinumab, have demonstrated substantial clinical efficacy in improving symptoms of moderate-to-severe PSO (6, 7). However, the extent to which these agents remodel systemic inflammatory networks *in vivo*, as well as the temporal dynamics of these changes, remains poorly characterized. Therefore, systematic investigation of the longitudinal changes in circulating inflammatory proteins during IL-17A inhibitor therapy is essential for elucidating the molecular mechanisms underlying therapeutic responses in PSO.

Proteins are central regulators of immune function and inflammatory signaling and are frequently perturbed under both pathological and therapeutic conditions. In immune-mediated inflammatory diseases such as PSO, dysregulated cytokines and immune-related proteins play critical roles in disease initiation, progression, and systemic manifestations by modulating immune activation, inflammatory cascades, and tissue homeostasis (1). Mass spectrometry–based proteomic approaches have been widely applied to quantitatively profile protein abundance and expression and have substantially contributed to the identification of disease-associated molecular signatures and therapeutic targets (8). Recently, Olink proteomics has become a sensitive platform for detecting low-abundance proteins that play a key role in the pathological process. This technology is based on highly sensitive and specific proximity extension assay (PEA), which only requires 1ul sample size, making it particularly well-suited for biomarker discovery in studies with limited clinical material (9). Olink proteomics has been applied to a broad range of immune and inflammation-related diseases, facilitating the identification of novel biomarkers and improving diagnostic performance (10–12). In addition, this method has been proven to be a stable and reliable protein quantitative method with good reproducibility and analytical stability (13, 14). To the best of our knowledge, a systematic and longitudinal characterization of plasma proteomic alterations during IL-17A inhibitor therapy in patients with PSO using Olink proteomics has not yet been reported.

In this study, we enrolled patients with PSO receiving secukinumab therapy and healthy controls and longitudinally collected plasma samples at baseline and at weeks 12 and 24 after treatment initiation. Olink Inflammation proteomics was used to systematically quantify a panel of inflammation- and immunity-related proteins, to identify potential biomarkers and treatment response–associated protein signatures correlated with clinical improvement.

## Materials and methods

### Study design and participants

**Figure 1:**
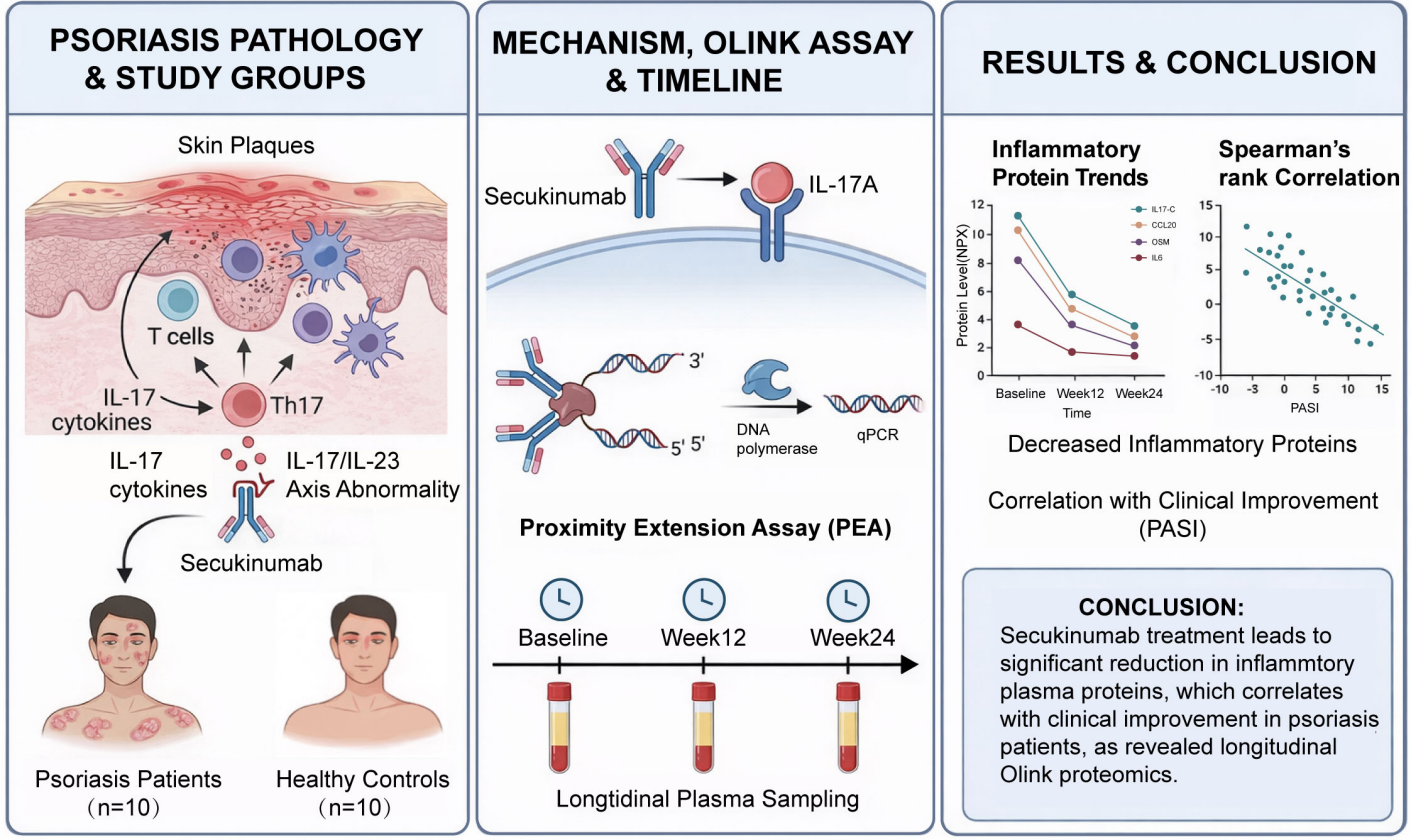
Workflow of longitudinal plasma proteomic profiling for biomarker discovery in psoriasis patients treated with secukinumab.

The overall research workflow diagram for this study is shown in **Figure 1**. This study was designed as a prospective longitudinal observational study. A total of 10 patients with moderate-to-severe plaque PSO who attended the Department of Dermatology at Ningxia Medical University General Hospital and received secukinumab therapy were enrolled, together with 10 age- and sex-matched healthy volunteers serving as the control group. All patients met the diagnostic criteria for plaque PSO according to the Chinese guidelines for the diagnosis and treatment of PSO and had not received any biologic therapy prior to enrollment. The main inclusion criteria were as follows: age ≥18 years, a clinical diagnosis of moderate-to-severe plaque PSO, eligibility for secukinumab treatment, and provision of written informed consent. The exclusion criteria included the presence of severe infections, autoimmune diseases, malignancies, or pregnancy, as well as recent use of systemic immunosuppressive medications.

All patients underwent clinical evaluation and plasma sample collection at baseline before treatment (T1), and at weeks 12 (T2) and 24 (T3) after treatment initiation. Plasma samples from NC were collected only once at the time of enrollment. The study protocol was approved by the Ethics Committee of Ningxia Medical University General Hospital (approval no. KYLL-2025-1773). Written informed consent was obtained from all participants, and the study was conducted in accordance with the principles of the Declaration of Helsinki.

### Treatment regimen and clinical assessment

All patients received the standard treatment regimen of secukinumab according to the prescribing information: an initial induction phase with subcutaneous injections of 300 mg at weeks 0, 1, 2, 3, and 4, followed by maintenance injections of 300 mg every 4 weeks. Clinical efficacy was assessed at time points T1, T2, and T3 using the Psoriasis Area and Severity Index (PASI), Body Surface Area (BSA), and Dermatology Life Quality Index (DLQI) as evaluation metrics.

### Plasma sample collection and processing

All blood samples were collected from peripheral veins into EDTA anticoagulant tubes in the early morning on an empty stomach. Plasma was separated by centrifugation at 1, 500 g for 10 minutes within 2 hours of collection. After aliquoting, plasma was immediately stored at −80 °C until centralized proteomic analysis. All samples were protected from repeated freeze-thaw cycles to minimize systematic errors introduced by sample handling.

### Olink proteomic profiling

Plasma samples were analyzed using the Olink Proximity Extension Assay technology, employing the Olink Target 96 Panel to quantitatively assess 92 inflammation-related proteins. Detailed information regarding these biomarkers is presented in **Supplementary Table 1**. The assay was performed by a qualified third-party laboratory following standard operating procedures. Protein expression levels were expressed as Normalized Protein Expression (NPX) values, representing log2-normalized relative quantitative values. Values below the limit of detection (LOD) were processed according to Olink recommended methodology. All samples were tested within the same batch to minimize batch effects. All protein data underwent missing value checks, LOD processing, and distribution checks. Proteins below the LOD were replaced with the half-minimum detectable value. Proteins present below the LOD in more than 50% of samples were excluded to ensure analytical reliability. All protein NPX values were used for subsequent statistical analysis.

### Statistical analysis

All statistical analyses were performed using R (version 4.5.2) (www.r-project.org). Continuous variables are presented as the mean ± standard deviation, and categorical variables as frequencies and percentages. Differences in protein expression between PSO and NC were assessed using nonparametric tests, with multiple comparisons adjusted for the false discovery rate (FDR) using the Benjamin–Hochberg procedure. To evaluate temporal changes in protein expression during treatment, an advanced Mfuzz analysis of DEPs was performed using the OmicStudio tools (15) available at https://www.omicstudio.cn/tool. Associations between protein expression (NPX) and clinical indices (PASI) were examined using Spearman’s rank correlation. For selected candidate biomarkers, logistic regression models were constructed to discriminate between PSO patients and NC. Model performance was evaluated using receiver operating characteristic (ROC) curves and the area under the curve (AUC). All statistical tests were two-tailed, and a P value < 0.05 was considered statistically significant.

## Results

### Cohort baseline characteristics

A total of 10 patients were included in the analysis, of whom 50.0% were male. The median age was 38 years (interquartile range [IQR], 29–56). At baseline, the median PASI score was 7.05 (IQR, 5.6–11.1), the median body surface area (BSA) involvement was 6.0% (IQR, 4.0–8.5), and the median Dermatology Life Quality Index (DLQI) score was 10 (IQR, 5–14). The median disease duration was 8.0 years (IQR, 4.0–15.0). Baseline demographic and clinical characteristics are summarized in **Table 1**, and further details are presented in **Supplementary Table 1**.

**Table 1:** Baseline characteristics of the participants.

Parameters	Value
Patients, n	10
Demographics	
Male/female, n (%)	5 (50.0)/5 (50.0)
Age, years, median [IQR]	38 [29–56]
Weight, kg, median [IQR]	72.0 [65.0–80.0]
Height, cm, median [IQR]	168.0 [162.0–174.0]
BMI, kg/m², median [IQR]	25.4 [23.1–27.8]
Family history of PSO	
Positive, n (%)	3 (30.0)
Negative, n (%)	6 (60.0)
Unknown, n (%)	2 (20.0)
Disease activity, median [IQR]	
PASI at baseline	7.05 [5.6–11.1]
BSA at baseline	6.0 [4.0–8.5]
DLQI at baseline	10 [5–14]
Disease duration, years	8.0 [4.0–15.0]
Co-morbidities, n (%)	
None	3 (30.0)
Psoriatic arthritis	2 (20.0)
Diabetes mellitus type II	2 (20.0)
Cardiovascular risk factor(s)	3 (30.0)
Depression/mental health disorder(s)	1 (10.0)
Immune-mediated disorder(s)	1 (10.0)
Current/past malignancies	0 (0.0)
Musculoskeletal disorder(s)	0 (0.0)

### Baseline plasma proteomic signature distinguishes PSO from NC

**Figure 2:**
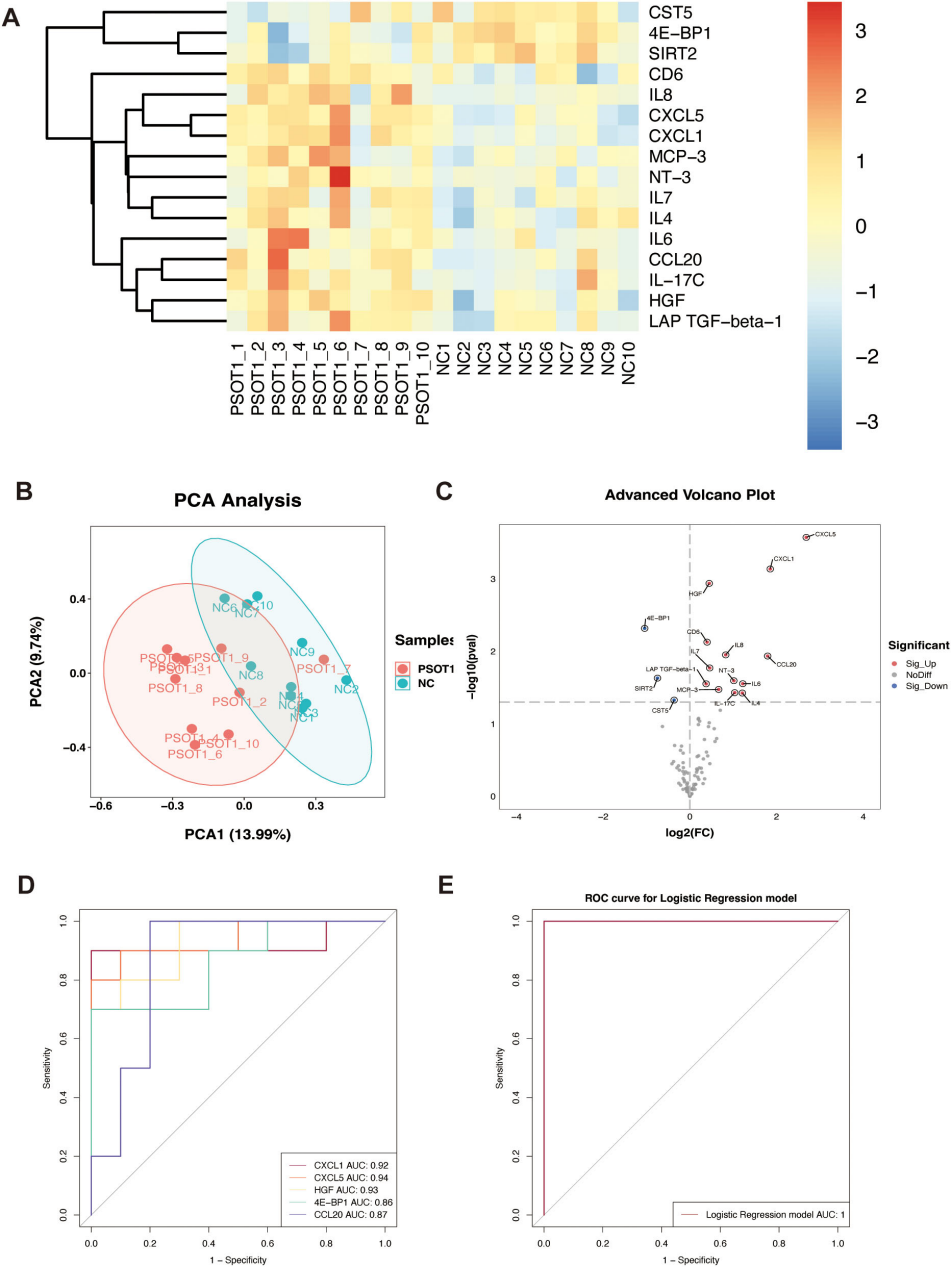
Identification of differentially expressed circulating proteins between patients with PSO and NC. **(A)** Heatmap of the significant DEPs. **(B)** Principal component analysis **(PCA)** based on the expression profiles of all quantified proteins, demonstrating a clear separation between PSO and NC. **(C)** Volcano plot illustrates the fold changes as well as P-values of inflammation-related biomarkers, with each point representing an individual protein. **(D)** ROC for the top diagnostic candidate proteins, illustrating their performance in discriminating PSO from NC samples. **(E)** ROC curve for the multivariable logistic regression model integrating selected proteins.

To assess the systemic inflammatory molecular profile of PSO, protein expression patterns were first compared between patient baseline plasma samples (T1) and NC. Heatmaps and PCA analysis based on differentially expressed proteins revealed distinct separation in protein expression patterns between PSO patients and NC (**Figures 2A, B**), indicating significant differences in systemic inflammatory protein profiles. Differential analysis further revealed that multiple inflammation- and immune-related proteins were significantly upregulated in PSO patients, including CXCL1, CXCL5, HGF, IL-8, CCL20, IL-6, IL-7, IL-4, IL-17C, NT-3, CD6, and MCP-3. Conversely, 4E-BP1, SIRT2, CST5, and LAP TGF-β1 were significantly downregulated in patients (**Figure 2C**). Evaluation of the diagnostic discriminatory power of individual proteins showed that the area under the receiver operating characteristic curve (AUC) for CXCL5, HGF, and CXCL1 was 0.94, 0.93, and 0.92, respectively; while CCL20 and 4E-BP1 yielded AUC values of 0.87 and 0.86, respectively (**Figure 2D**). A logistic regression model incorporating multiple differentially expressed proteins was constructed to distinguish PSO patients from NC, achieving an AUC of 1.00 in the study cohort (**Figure 2E**).

### Functional enrichment analyses of DEPs

**Figure 3:**
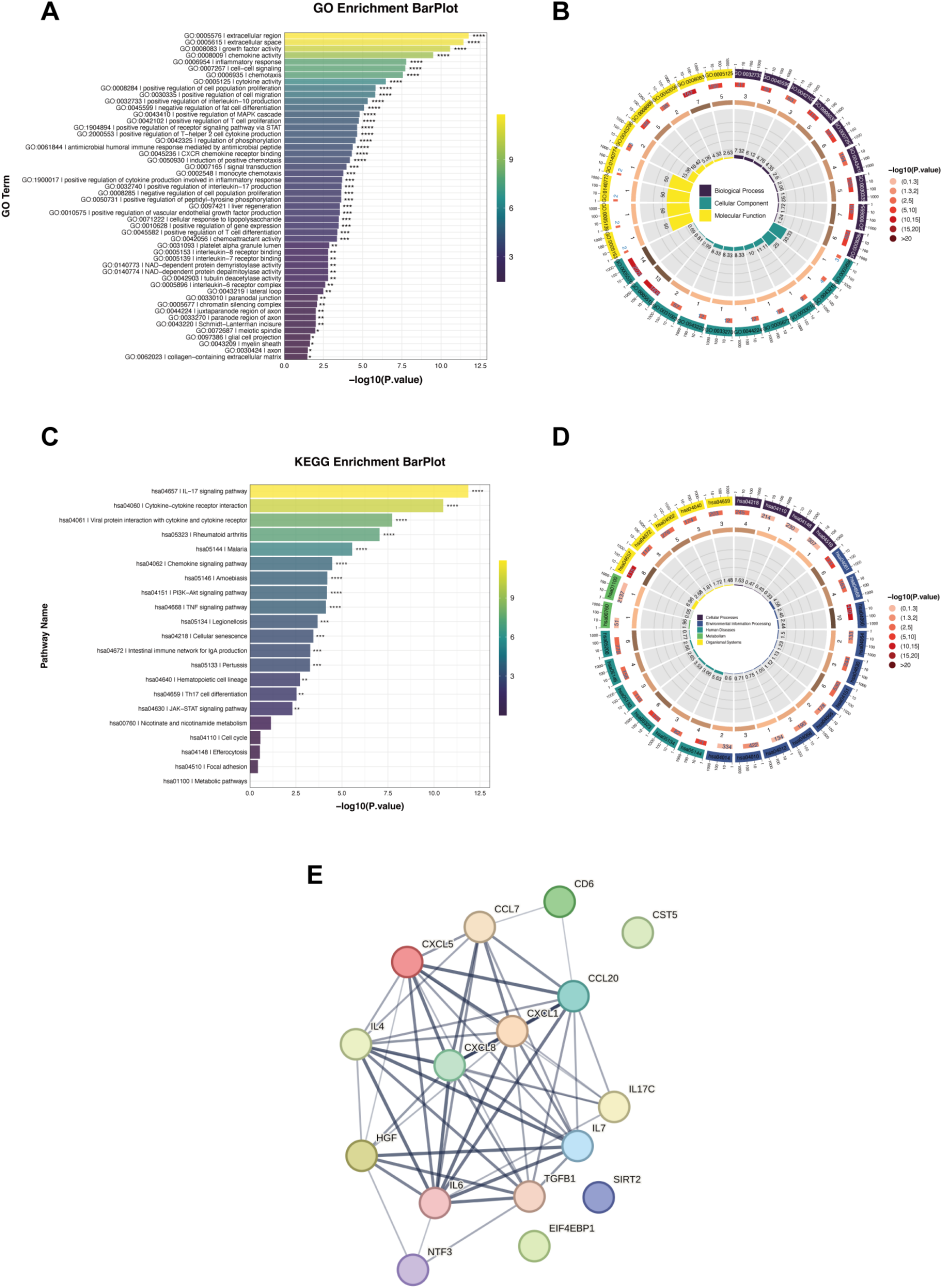
Functional enrichment and interaction network analysis of DEPs between the PSO and NC groups. **(A)** Bar plot showing the top enriched GO terms for biological processes, cellular component, and molecular function ranked by −log10 (P value). **(B)** Circular visualization of GO enrichment results. **(C)** Bar plot of the top enriched KEGG pathways, ranked by −log10 (P value). **(D)** Circular representation of KEGG pathway enrichment results. **(E)** PPI network constructed based on the significantly altered proteins, where nodes represent individual proteins and edges indicate interactions. Node color reflects relative expression change, and edge thickness corresponds to interaction confidence.

Gene Ontology (GO) enrichment analysis revealed that at the cellular component (CC) level, these proteins were mainly localized to the extracellular region and extracellular space; At the molecular function (MF) level, they were enriched in chemokine activity, growth factor activity, and cytokine receptor binding; at the biological process (BP) level, they were significantly enriched in inflammatory response, immune cell chemotaxis, cytokine production and signaling, T cell differentiation, and Th17-related immune regulation (**Figures 3A, B**). Kyoto Encyclopedia of Genes and Genomes (KEGG) pathway enrichment analysis revealed that differentially expressed proteins were predominantly enriched in inflammation and immune-related signaling pathways (**Figures 3C, D**). Among these, the IL-17 signaling pathway and cytokine-cytokine receptor interaction pathway exhibited the highest enrichment levels. Additionally, chemokine signaling pathways, TNF signaling pathways, PI3K-Akt signaling pathways, and JAK-STAT signaling pathways were significantly enriched, suggesting that PSO-associated differentially expressed proteins primarily participate in systemic inflammatory responses, immune signal transduction, and cytokine-mediated regulatory processes.

Protein–protein interaction (PPI) network analysis revealed that chemokines and inflammatory cytokines represented by CXCL1, CXCL5, CXCL8, CCL20, IL6, IL7, and HGF formed a highly interconnected core module (**Figure 3E**). In contrast, proteins such as TGFB1, SIRT2, EIF4EBP1, CST5, and NTF3 were located at the periphery of the network with lower connectivity, potentially participating more in regulatory or secondary processes.

### Correlative analyses of DEPs

**Figure 4:**
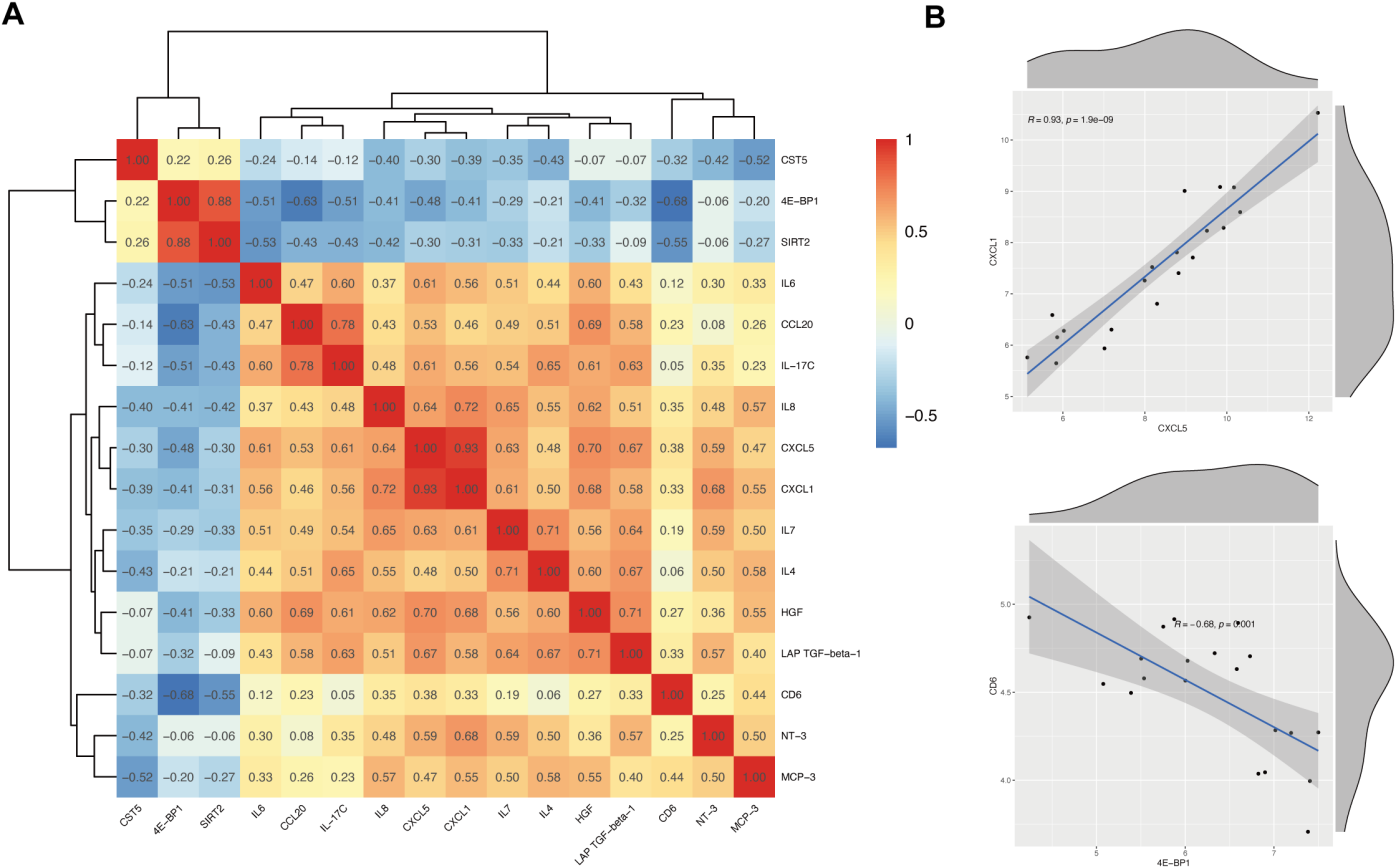
Correlation analysis of DEPs between the PSO and NC groups. **(A)** Heatmap showing pairwise correlations among the DEPs identified between the PSO and NC groups. Correlation coefficients were calculated using Spearman’s rank correlation method. Red, positively related; blue, negatively related. **(B)** Representative scatterplots illustrating the relationships between selected pairs of DEPs with relatively strong correlations.

To explore the synergistic relationships among differentially expressed proteins, we performed pairwise correlation analyses on the plasma levels of significantly differentiated proteins at baseline. Overall, most inflammatory and chemokine proteins exhibited a significant positive clustering pattern, while metabolic regulation-related proteins showed a negative correlation trend with the inflammatory module (**Figure 4A**). This suggests that these proteins may reflect distinct yet interconnected biological processes at the systems level. Concurrently, IL-17C, CCL20, IL-6, IL-8, CXCL1, and CXCL5 collectively form a positively correlated module centered on the Th17-inflammation axis. Within this module, IL-17C exhibits moderate to strong positive correlations with CCL20, IL-6, CXCL1, and CXCL5, indicating high co-variation among these inflammation- and immune chemokine-related proteins at the circulatory level. Furthermore, HGF exhibits significant positive correlations with multiple inflammation-related proteins, suggesting HGF may synchronously modulate with heightened inflammatory activity and tissue response processes. Specifically, chemokines CXCL1 and CXCL5 exhibited an extremely strong positive correlation (R = 0.93, p = 1.9 × 10^-9^), suggesting highly synergistic regulation between them in PSO patients. In contrast, 4E-BP1 exhibits systematic negative correlations with the inflammatory module, particularly showing a significant negative correlation with CD6 (R = −0.68, p = 0.001), indicating that its directional changes are opposite to those of the inflammatory chemotaxis pathway (**Figure 4B**).

### Time-resolved proteomic changes following secukinumab treatment

**Figure 5:**
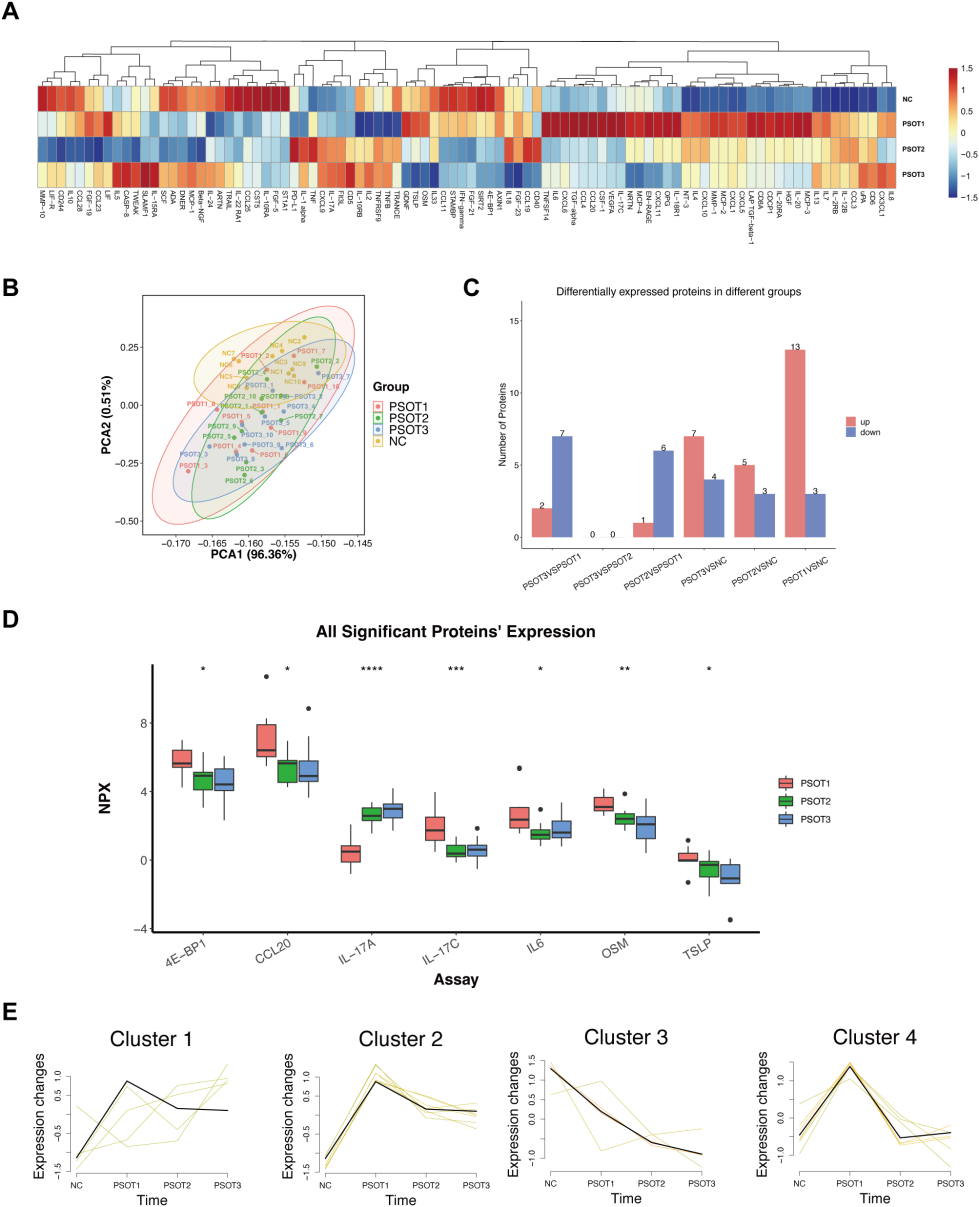
Inflammation-related plasma proteomic profiles across PSO and NC groups. **(A)** Heatmap showing the overall expression levels of inflammation-related proteins across NC, PSOT1, PSOT2, and PSOT3 samples. **(B)** PCA illustrating the separation among the four groups. **(C)** Bar plot showing the number of DEPs in pairwise comparisons between groups. **(D)** Box plots showing the expression levels of selected significant proteins across PSOT1, PSOT2, and PSOT3 groups. **(E)** Mfuzz clustering analysis identifying four typical temporal expression patterns of proteins across NC, PSOT1, PSOT2, and PSOT3.

Distinct expression patterns of inflammation-related proteins were observed across the four groups, including healthy controls (NC) and psoriasis patients at baseline (PSOT1) and at weeks 12 (PSOT2) and 24 (PSOT3) after secukinumab initiation (**Figures 5A, B**). The heatmap reveals distinct proteomic differences between NC and PSOT1, with multiple protein clusters showing opposite or markedly divergent expression levels. Notably, the expression patterns in PSOT2 and PSOT3 gradually shifted toward those observed in the NC group, indicating a progressive normalization of the circulating proteomic profile during treatment. **Figure 5C** summarizes the number of DEPs identified between each pair of groups. The largest number of proteomic differences occurred between untreated psoriasis patients and NC, whereas relatively few differences were observed among treatment time points, indicating that treatment progressively attenuated disease-associated proteomic alterations.

To visualize longitudinal changes in representative differentially expressed proteins at the individual level, plasma expression levels of proteins showing significant differences across multiple groups—4E-BP1, CCL20, IL-17A, IL-17C, IL-6, OSM, and TSLP—were visualized at three time points (T1, T2, T3). Results showed that expression levels of 4E-BP1, CCL20, IL-17C, IL-6, OSM, and TSLP decreased from T1 to T2 and further declined at T3; conversely, IL-17A increased from T1 to T2 and maintained elevated levels at T3 (**Figure 5D**). To further characterize global temporal expression patterns, Mfuzz clustering analysis was performed on all differentially expressed proteins, identifying four distinct temporal expression trends (**Figure 5E**). Cluster 1, Cluster 2 and Cluster 4 exhibited early downregulation followed by partial stabilization, whereas Cluster 3 showed a continuous decrease throughout the treatment period.

### Association between longitudinal proteomic changes and clinical response

**Figure 6:**
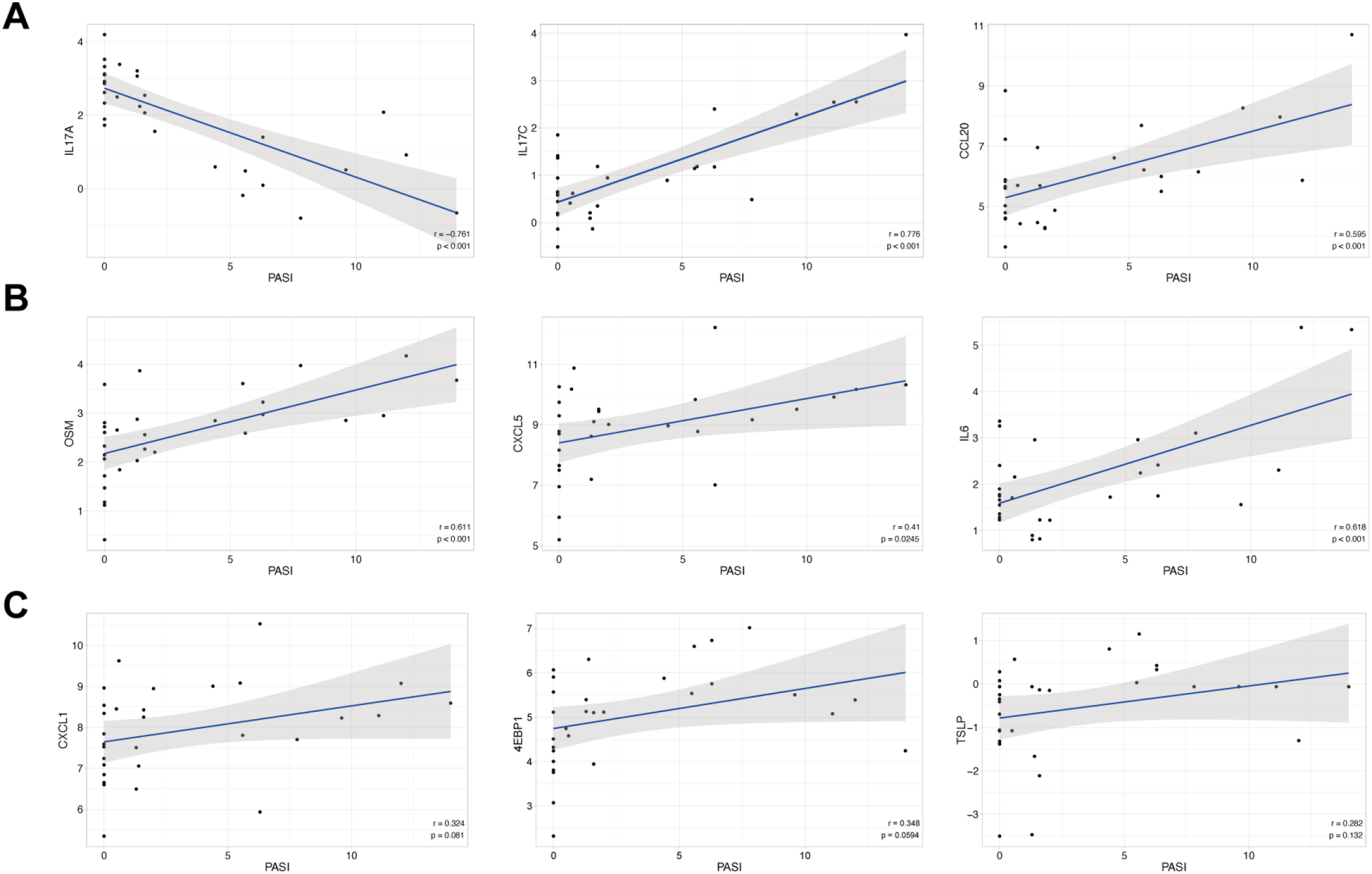
Correlative analyses between circulating protein levels and PASI in PSO. **(A)** Correlations between PASI and IL-17A, IL-17C, and CCL20. **(B)** Correlations between PASI and OSM, CXCL5, and IL-6. **(C)** Correlations between PASI and CXCL1, 4E-BP1, and TSLP.

To assess the relationship between circulating protein levels and disease severity, Spearman’s rank correlation analysis was performed between baseline PASI scores and the expression levels of differentially expressed proteins. Several proteins showed significant correlations with PASI. Specifically, IL-17A levels were negatively correlated with PASI, whereas IL-17C, CCL20, OSM, CXCL5, and IL-6 exhibited significant positive correlations with PASI (**Figures 6A–C**). These findings indicate that higher expression levels of these inflammatory and chemotactic mediators are associated with greater disease severity. In contrast, no significant correlations were observed between PASI and the remaining proteins analyzed.

### ELISA validation of diagnostic and treatment-responsive proteins

**Figure 7:**
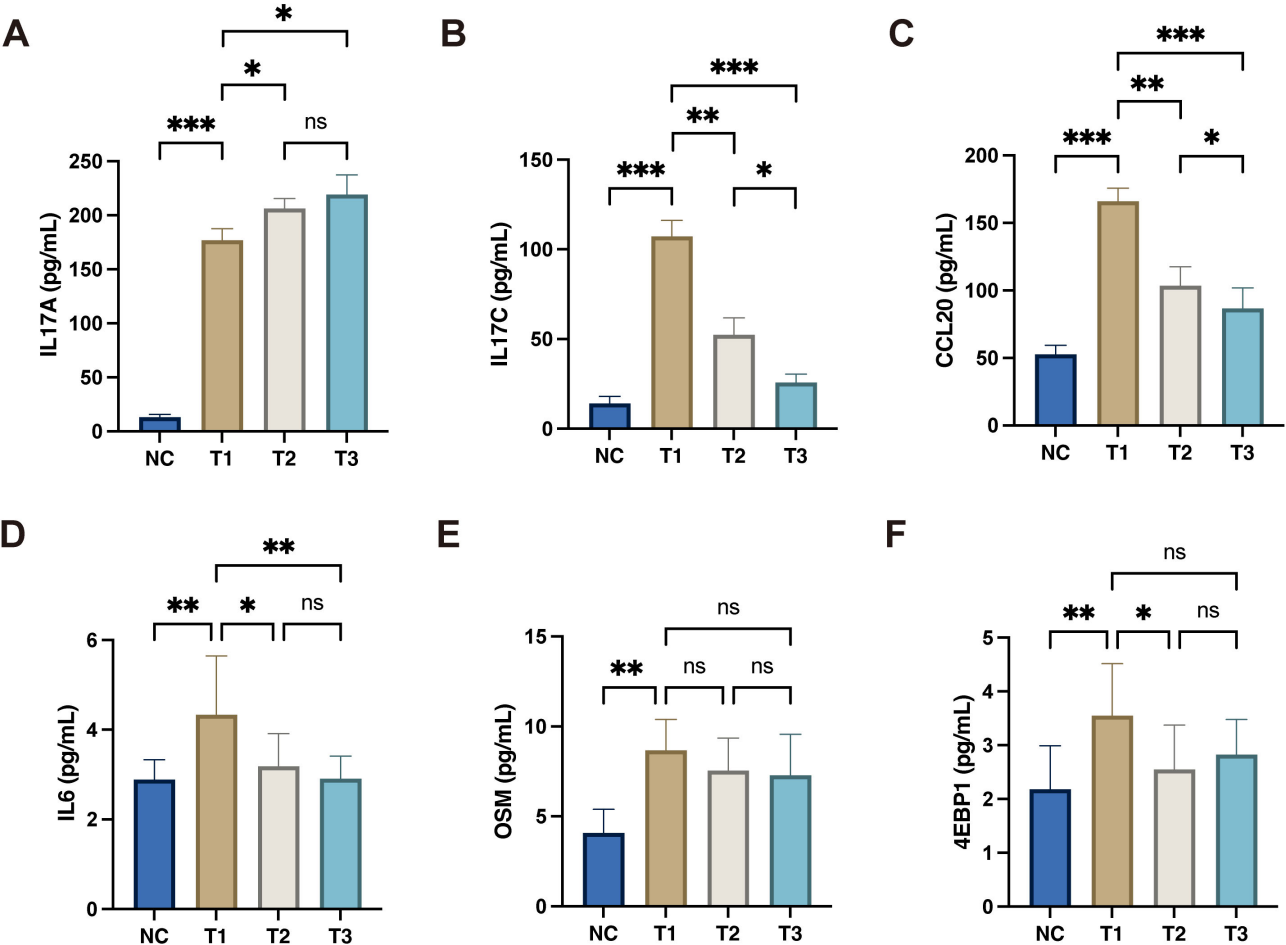
ELISA validation of longitudinal changes in plasma inflammatory proteins during secukinumab treatment. **(A)** Plasma concentration of IL17A in NC and treatment time points. **(B)** Plasma concentration of IL17C in NC and treatment time points. **(C)** Plasma concentration of CCL20 in NC and treatment time points. **(D)** Plasma concentration of IL6 in NC and treatment time points. **(E)** Plasma concentration of OSM in NC and treatment time points. **(F)** Plasma concentration of 4EBP1 in NC and treatment time points. *p < 0.05, **p < 0.01, ***p < 0.001.

To validate the Olink proteomics findings, this study further employed ELISA to detect circulating levels of 4E-BP1, CCL20, IL-17A, IL-17C, IL-6, and OSM at different treatment time points (T1, T2, T3). Results showed that compared with pretreatment levels, IL-6, IL-17C, and CCL20 significantly decreased after treatment and remained at lower levels at subsequent time points (**Figures 7B–D**). IL-17A levels significantly increased from T1 to T2, while no statistically significant difference was observed between T2 and T3 (**Figure 7A**). OSM also showed a downward trend post-treatment but failed to reach statistical significance (**Figure 7E**). In contrast, 4E-BP1 exhibited lower concentrations and greater variability in peripheral blood, with no significant differences observed across different time points (**Figure 7F**).

## Discussion

PSO is a chronic immune-mediated inflammatory skin disease primarily characterized by hyperplasia of keratinocytes and the formation of scaly plaques on the skin surface (16). Approximately 2% of the global population is affected by PSO, with plaque PSO accounting for 90% of all cases (17). PSO not only causes significant physical damage and reduced quality of life for patients but also imposes a substantial social and economic burden, making it a major global health issue (18). The immunopathological mechanisms of PSO are complex, with its core feature being an inflammatory amplification cycle driven by the synergistic dysregulation of innate and adaptive immune responses. This involves the activation of T cells, particularly Th1/Th17 cells, whose release of multiple cytokines—such as IL-17A, TNF-α, IFN-γ, and IL-22—serves as key drivers of the local inflammatory response in PSO (4).

With advancing research, traditional treatment strategies—including topical corticosteroids, phototherapy, and systemic medications like methotrexate and cyclosporine—have gradually been replaced by biologics (19). Particularly, biologics targeting IL-17A and TNF-α have demonstrated significant efficacy in treating moderate-to-severe PSO (20). The application of biologics marks the advent of precision targeted therapy in PSO management. Current drugs primarily target IL-12, IL-17, IL-23, TNF-α, and their receptors (2). Secukinumab, the first fully human monoclonal antibody to inhibit immune responses by neutralizing IL-17A, has become one of the preferred biologics for treating moderate-to-severe PSO since its approval for PSO treatment in 2015 (21–23). This study utilized Olink plasma proteomics technology to identify circulating protein signatures distinguishing PSO patients from healthy individuals and characterized the dynamic changes in inflammation- and immunity-related proteins during secukinumab therapy.

In this study, CXCL1 and CXCL5 reliably distinguished PSO patients from NC at baseline and were validated in an independent ELISA cohort, suggesting their potential as circulating diagnostic biomarkers. Previous studies indicate that CXCL1/CXCL5, as typical CXC chemokines, are induced in keratinocytes and myeloid cells under the IL-17–NF-κB axis (24, 25). They participate in neutrophil recruitment and inflammatory amplification, forming a crucial component of PSO’s “neutrophil–keratinocyte” positive feedback loop (4). Given that psoriatic lesions pathologically feature Munro microabscesses and neutrophil infiltration, the detectable circulating differences in these chemokines are biologically plausible (26). Accordingly, relative to upstream cytokines with predominantly localized paracrine activity, these chemokines may be more informative as plasma-level indicators of disease presence in a biomarker context. From a biomarker application perspective, the concurrent reflection of core pro-inflammatory pathway activation and relatively stable circulating output characteristics of CXCL1/CXCL5 explains their robust discriminative power even with limited sample sizes (27). This supports their prioritization for subsequent large-scale validation and multi-biomarker combination analysis.

Unlike CXCL1 and CXCL5, which primarily reflect neutrophil chemotaxis and inflammatory amplification, CCL20 and HGF are more likely to indicate distinct pathogenic processes in PSO (28, 29). Specifically, CCL20 reflects the maintenance of the Th17 cell migration circuit (30, 31). As the specific ligand for CCR6, CCL20 mediates the recruitment of CCR6^+^ Th17 and Tc17 cells to inflamed skin and synergizes with IL-17 signaling to form a key migration–effector module that sustains local inflammatory responses (32). This “CCL20–CCR6–Th17” axis is therefore considered to be closely associated with persistent inflammatory activity and lesion expansion in PSO (33, 34). Accordingly, differences in circulating CCL20 not only have potential diagnostic value but may also serve as a dynamic indicator of immune activation and inflammatory cell trafficking (35). In contrast, HGF predominantly reflects processes related to tissue repair, angiogenesis, and inflammation-associated microenvironment remodeling (36). Previous studies have shown that the HGF/c-MET signaling pathway regulates keratinocyte proliferation and migration, vascular responses, and the construction of the inflammatory microenvironment (37). The plasma HGF differences observed in this study thus suggest that HGF may function as a complementary circulating biomarker reflecting tissue and vascular reactivity. Notably, a recent case report using Olink proteomic profiling in a patient with generalized pustular psoriasis treated with an IL-17A inhibitor (ixekizumab) demonstrated significant downregulation of HGF and several inflammation-related proteins after treatment (38). Although derived from a single case, these findings are in line with our results and provide additional support for the biological relevance of IL-17A–mediated pathway modulation. Together with chemokine-based markers, this complementarity may enhance the ability of multi-biomarker models to capture the heterogeneity of PSO and improve their biological interpretability.

Among all proteins showing treatment-associated changes, IL-17C levels were significantly correlated with the magnitude of PASI improvement. Primarily produced by epithelial cells, IL-17C amplifies local inflammatory responses through autocrine and paracrine signaling (39). Its reduction may therefore more directly reflect attenuation of epidermal immune activation. These findings suggest that IL-17C could serve as a circulating biomarker for dynamic monitoring of treatment response, complementing the limitations of static diagnostic markers in efficacy assessment (40). Notably, despite being the direct therapeutic target of secukinumab, circulating IL-17A levels increased during treatment. This phenomenon can be explained by the pharmacokinetic properties of the drug (41, 42). Antibody binding stabilizes circulating IL-17A, reducing its receptor-mediated clearance and tissue uptake (43). A recent study in patients with spondyloarthritis (SpA) provides more direct evidence for this phenomenon: serum IL-17A was measured at baseline and after 6 and 12 months of secukinumab treatment, and an IgG column–based separation assay was used to distinguish free IL-17A from antibody-conjugated (secukinumab-bound) IL-17A (44). Results showed a significant increase in serum IL-17A post-treatment, primarily driven by the secukinumab-bound fraction, while free IL-17A levels remained relatively stable. More importantly, elevated circulating IL-17A levels negatively correlated with disease activity measures, suggesting that IL-17A “accumulation” may actually correlate with improved clinical response. This study therefore proposes that the observed increase in IL-17A during treatment primarily reflects the formation of antibody-complexes and their accumulation in circulation—a phenotype of effective target blockade—rather than heightened IL-17 pathway activity or worsening inflammation. Based on this, we propose that when interpreting biomarker dynamics associated with targeted therapies, it is crucial to distinguish between quantitative changes and functional suppression, the apparent increase in circulating IL-17A may primarily represent biologically inactive, bound cytokines and does not necessarily indicate reactivation or enhancement of IL-17 functional pathways.

Additionally, this study observed that levels of 4E-BP1, CCL20, IL-17C, IL-6, OSM, and TSLP decreased by week 12 of treatment and declined further by week 24. This temporal pattern is consistent with the clinical profile of IL-17A inhibitors, characterized by an early onset of action followed by sustained consolidation, and indicates a progressive attenuation of inflammatory activity at the circulating level (45). From a pathway perspective, CCL20 and IL-6/OSM correspond to the Th17 cell migration–maintenance module and the pro-inflammatory cytokine amplification module, respectively (46). Following blockade of the IL-17 pathway, inflammatory transcriptional programs in keratinocytes are suppressed, leading to downregulation of downstream chemokine and cytokine networks (47), as reflected by coordinated reductions in CCL20, IL-6, and OSM. TSLP, an epithelial-derived alarmin linking barrier stress to immune polarization, also declined during treatment, suggesting alleviation of inflammatory stress signaling in the skin barrier and a reduction in the overall inflammatory burden (48). Notably, 4E-BP1, a key regulator within the mTOR-associated translational control axis, also showed decreased expression. This change likely reflects suppression of keratinocyte proliferation, metabolic activity, and protein synthesis programs, rather than merely a reduction in immune cytokine signaling (49). Together, this pattern is consistent with the pathological framework of coupled immune inflammation and keratinocyte hyperproliferation in PSO and indicates that the circulating proteome can capture multilevel responses ranging from immune signaling to tissue-level effects (50). Overall, the consistent decline of these proteins at week 12 and their further reduction at week 24 support their potential utility as biomarkers for therapeutic process monitoring, reflecting sustained attenuation of downstream effector networks following IL-17A blockade.

This study has certain limitations. The sample size was small and derived from a single-center cohort, and all subjects were patients who responded well to secukinumab treatment, making it impossible to assess the molecular characteristics of non-responders or low responders. In addition, the follow-up duration was limited to 24 weeks, limiting our ability to assess longer-term durability or secondary loss of response. Although multiple testing corrections and internal statistical control strategies were employed during analysis, the identified diagnostic and efficacy-related proteins require further validation in larger sample sizes and independent external cohorts, and through complementary experimental approaches. This study primarily relies on peripheral plasma proteomics data and cannot directly reflect molecular changes within the local microenvironment of skin lesions or specific immune cell subsets. Future studies with larger cohorts and integrated transcriptomic analyses are needed to further validate and strengthen these findings. Despite these limitations, the research systematically depicts the dynamic evolution of molecular profiles during IL-17A inhibition therapy from a time-resolved proteomics perspective. It provides an analytical framework for integrating longitudinal molecular characteristics with clinical efficacy to identify potential biomarkers.

## Conclusions

This study systematically identified plasma protein signatures distinguishing PSO patients from healthy individuals and revealed coordinated changes in circulating immune and inflammatory proteins during secukinumab therapy. Multiple inflammatory and chemokine factors showed consistent downregulation following IL-17A inhibition, reflecting systemic regulation of the cytokine network. Functional enrichment analysis indicated that differentially expressed proteins primarily participated in cytokine-receptor interactions and IL-17-related signaling pathways, highlighting the critical role of these immune pathways in PSO pathogenesis and treatment response. Additionally, IL-17C was identified as a potential circulating biomarker associated with clinical improvement. To our knowledge, this represents the first longitudinal plasma proteomics analysis of secukinumab treatment for PSO using the Olink platform, providing potential circulating biomarkers for disease identification and treatment monitoring in PSO.

## Data Availability

The raw data supporting the conclusions of this article will be made available by the authors, without undue reservation.
