# Supplementary material for: Longitudinal plasma proteomics identifies diagnostic and response-associated inflammatory and immune biomarkers in psoriasis following secukinumab therapy
Source: Front Immunol. 2026 Apr 24;17:1806248. doi: 10.3389/fimmu.2026.1806248 (PMC13153052; doi:10.3389/fimmu.2026.1806248)
Supplement: Supplementary file 1 [file SupplementaryFile1.docx]

**Supporting Information :**

Information of 92 inflammation-related proteins in Olink analysis (Table S1)
Demographics and clinical characteristics of the participants (Table S2)

Raw data of the Olink proteomics analysis (XLSX)
Raw data of the ELISA analysis (XLSX)

Table S1. Detailed information of 92 inflammaiton-related proteins

| **Protein name** | **UniProt ID** | **Gene** |
| --- | --- | --- |
| Tumor necrosis factor receptor superfamily member 11B | O00300 | TNFRSF11B |
| C-X-C motif chemokine 11 | O14625 | CXCL11 |
| Tumor necrosis factor ligand superfamily member 11 | O14788 | TNFSF11 |
| Axin-1 | O15169 | AXIN1 |
| C-C motif chemokine 25 | O15444 | CCL25 |
| Tumor necrosis factor ligand superfamily member 12 | O43508 | TNFSF12 |
| Tumor necrosis factor ligand superfamily member 14 | O43557 | TNFSF14 |
| STAM-binding protein | O95630 | STAMBP |
| Fibroblast growth factor 19 | O95750 | FGF19 |
| Interleukin-33 | O95760 | IL33 |
| Urokinase-type plasminogen activator | P00749 | PLAU |
| Adenosine deaminase | P00813 | ADA |
| Protransforming growth factor alpha | P01135 | TGFA |
| Transforming growth factor beta-1 proprotein | P01137 | TGFB1 |
| Beta-nerve growth factor | P01138 | NGF |
| Lymphotoxin-alpha | P01374 | LTA |
| Tumor necrosis factor | P01375 | TNF |
| Interferon gamma | P01579 | IFNG |
| Interleukin-1 alpha | P01583 | IL1A |
| T-cell surface glycoprotein CD8 alpha chain | P01732 | CD8A |
| C-X-C motif chemokine 10 | P02778 | CXCL10 |
| Interstitial collagenase | P03956 | MMP1 |
| Interleukin-4 | P05112 | IL4 |
| Interleukin-5 | P05113 | IL5 |
| Interleukin-6 | P05231 | IL6 |
| T-cell surface glycoprotein CD5 | P06127 | CD5 |
| Stromelysin-2 | P09238 | MMP10 |
| Growth-regulated alpha protein | P09341 | CXCL1 |
| Macrophage colony-stimulating factor 1 | P09603 | CSF1 |
| Interleukin-8 | P10145 | CXCL8 |
| C-C motif chemokine 3 | P10147 | CCL3 |
| Fibroblast growth factor 5 | P12034 | FGF5 |
| Interleukin-7 | P13232 | IL7 |
| C-C motif chemokine 4 | P13236 | CCL4 |
| C-C motif chemokine 2 | P13500 | CCL2 |
| Oncostatin-M | P13725 | OSM |
| Hepatocyte growth factor | P14210 | HGF |
| Interleukin-2 receptor subunit beta | P14784 | IL2RB |
| Leukemia inhibitory factor | P15018 | LIF |
| Vascular endothelial growth factor A, long form | P15692 | VEGFA |
| Neurotrophin-3 | P20783 | NTF3 |
| Kit ligand | P21583 | KITLG |
| Interleukin-10 | P22301 | IL10 |
| Tumor necrosis factor receptor superfamily member 5 | P25942 | CD40 |
| Cystatin-D | P28325 | CST5 |
| Interleukin-12 subunit beta | P29460 | IL12B |
| T-cell differentiation antigen CD6 | P30203 | CD6 |
| Interleukin-13 | P35225 | IL13 |
| Glial cell line-derived neurotrophic factor | P39905 | GDNF |
| Leukemia inhibitory factor receptor | P42702 | LIFR |
| C-X-C motif chemokine 5 | P42830 | CXCL5 |
| Fms-related tyrosine kinase 3 ligand | P49771 | FLT3LG |
| Sulfotransferase 1A1 | P50225 | SULT1A1 |
| Tumor necrosis factor ligand superfamily member 10 | P50591 | TNFSF10 |
| Eotaxin | P51671 | CCL11 |
| C-C motif chemokine 23 | P55773 | CCL23 |
| Interleukin-2 | P60568 | IL2 |
| Fractalkine | P78423 | CX3CL1 |
| C-C motif chemokine 20 | P78556 | CCL20 |
| C-C motif chemokine 8 | P80075 | CCL8 |
| C-C motif chemokine 7 | P80098 | CCL7 |
| C-X-C motif chemokine 6 | P80162 | CXCL6 |
| Protein S100-A12 | P80511 | S100A12 |
| Tumor necrosis factor receptor superfamily member 9 | Q07011 | TNFRSF9 |
| C-X-C motif chemokine 9 | Q07325 | CXCL9 |
| Interleukin-10 receptor subunit beta | Q08334 | IL10RB |
| Interleukin-24 | Q13007 | IL24 |
| Interleukin-15 receptor subunit alpha | Q13261 | IL15RA |
| Signaling lymphocytic activation molecule | Q13291 | SLAMF1 |
| Interleukin-18 receptor 1 | Q13478 | IL18R1 |
| Eukaryotic translation initiation factor 4E-binding protein 1 | Q13541 | EIF4EBP1 |
| Interleukin-10 receptor subunit alpha | Q13651 | IL10RA |
| Interleukin-18 | Q14116 | IL18 |
| Caspase-8 | Q14790 | CASP8 |
| Interleukin-17A | Q16552 | IL17A |
| Artemin | Q5T4W7 | ARTN |
| NAD-dependent protein deacetylase sirtuin-2 | Q8IXJ6 | SIRT2 |
| Interleukin-22 receptor subunit alpha-1 | Q8N6P7 | IL22RA1 |
| Delta and Notch-like epidermal growth factor-related receptor | Q8NFT8 | DNER |
| Thymic stromal lymphopoietin | Q969D9 | TSLP |
| C-C motif chemokine 13 | Q99616 | CCL13 |
| C-C motif chemokine 19 | Q99731 | CCL19 |
| Neurturin | Q99748 | NRTN |
| Natural killer cell receptor 2B4 | Q9BZW8 | CD244 |
| Fibroblast growth factor 23 | Q9GZV9 | FGF23 |
| CUB domain-containing protein 1 | Q9H5V8 | CDCP1 |
| C-C motif chemokine 28 | Q9NRJ3 | CCL28 |
| Fibroblast growth factor 21 | Q9NSA1 | FGF21 |
| Interleukin-20 | Q9NYY1 | IL20 |
| Programmed cell death 1 ligand 1 | Q9NZQ7 | CD274 |
| Interleukin-17C | Q9P0M4 | IL17C |
| Interleukin-20 receptor subunit alpha | Q9UHF4 | IL20RA |

Table S2. Demographics and clinical characteristics of the participants.

| **Parameters** | **Value** |
| --- | --- |
| **Patients,n** | 10 |
| **Demographics** |  |
| Male/female, n (%) | 5 (50.0)/5 (50.0) |
| Age, years, median [IQR] | 38 [29–56] |
| Weight, kg, median [IQR] | 72.0 [65.0–80.0] |
| Height, cm, median [IQR] | 168.0 [162.0–174.0] |
| BMI, kg/m², median [IQR] | 25.4 [23.1–27.8] |
| **Family history of psoriasis** |  |
| Positive, n (%) | 3 (30.0) |
| Negative, n (%) | 6 (60.0) |
| Unknown, n (%) | 2 (20.0) |
| **Disease activity, median [IQR]** |  |
| PASI at baseline | 7.05 [5.6–11.1] |
| BSA at baseline | 6.0 [4.0–8.5] |
| DLQI at baseline | 10 [5–14] |
| Disease duration, years | 8.0 [4.0–15.0] |
| **Smoking status, n (%)** |  |
| Active smoking | 4 (40.0) |
| Previous smoking | 2 (20.0) |
| Never smoking | 4 (40.0) |
| **Co-morbidities, n (%)** |  |
| None | 3 (30.0) |
| Psoriatic arthritis | 2 (20.0) |
| Diabetes mellitus type II | 2 (20.0) |
| Cardiovascular risk factor(s) | 3 (30.0) |
| Depression / mental health disorder(s) | 1 (10.0) |
| Immune-mediated disorder(s) | 1 (10.0) |
| Current/past malignancies | 0 (0.0) |
| Musculoskeletal disorder(s) | 0 (0.0) |
| **Concomitant medication, n (%)** |  |
| None | 4 (40.0) |
| Other immunosuppressantsᵃ | 0 (0.0) |
| Antidiabetic | 1 (10.0) |
| Antihypertensive | 3 (30.0) |
| β-blocker | 1 (10.0) |
| Antihyperlipidemic agent | 2 (20.0) |
| Antidepressant | 1 (10.0) |
| Antiarrhythmic | 0 (0.0) |
| **Previous treatments** |  |
| Non-biologic, n (%) | 8 (80.0) |
| Methotrexate | 6 (60.0) |
| Cyclosporin | 5 (50.0) |
| Retinoids | 3 (30.0) |
